# Bleeding From Left Atrial Appendage During Atrial Septal Defect Closure Through Right Anterolateral Thoracotomy: An Unusual Complication

**DOI:** 10.7759/cureus.34138

**Published:** 2023-01-24

**Authors:** Satyapriya Mohanty, Arvind Pandey, Firdoos Ahmad Mir, Anindya Banerjee, Debasish Das

**Affiliations:** 1 Cardiothoracic Surgery, All India Institute of Medical Sciences, Bhubaneswar, IND; 2 Cardiothoracic Surgery, Banaras Hindu University Institute of Medical Science (BHUIMS), Banaras, IND; 3 Cardiothoracic Surgery, Sher-i- Kashmir Institute of Medical Sciences, Srinagar, IND; 4 Cardiology, All India Institute of Medical Sciences, Bhubaneswar, IND

**Keywords:** phrenic nerve injury, left atrial appendage, bleeding, surgery, atrial septal defect

## Abstract

Anterolateral thoracotomy is frequently used for the closure of ostium secundum atrial septal defect (ASD). The cosmetic result has become an important feature. There are various complications of anterolateral thoracotomy like persistent postoperative pain, phrenic nerve injury, atelectasis, and bleeding. We report a case of ASD closure through anterolateral thoracotomy who had bleeding of the left atrial appendage (LAA), which is an unusual and rare complication.

## Introduction

Right anterolateral thoracotomy is frequently used for the closure of ostium secundum atrial septal defect (ASD) which is a cosmetic and minimally invasive procedure usually done for young female and obese patients. Notable complications during anterolateral thoracotomy include phrenic nerve palsy, atelectasis of the right lung, and bleeding from the access site [1]. Bleeding from the left atrial appendage (LAA) is a rare phenomenon during right anterolateral thoracotomy. Atrial appendages are thin paper-like structures that get an injury from trivial unnoticed trauma during the procedure of the aortic cross-clamp. Those unnoticed trauma lead to catastrophic intra and post-operative bleeding and cardiac tamponade which requires urgent reexploration adding to the morbidity and mortality of the surgical procedure.

We report a case of a five-year-old child with ostium secundum ASD not amenable to device closure and was planned for minimally invasive surgical ASD closure. After completion of the procedure, it was noticed that there was a small tear in the LAA which we repaired immediately on cardiopulmonary bypass (CPB) and fibrillating the heart without cardioplegia. The injury to the LAA was caused by the placement of the conventional vascular clamp used for the aortic cross-clamp. Repair of the LAA injury was facilitated by the placement of a Chitwood atraumatic vascular clamp used for aortic cross-clamp inserted through the right second intercostal space and the injured LAA was held with Cooley's atraumatic vascular clamp. Our case is an extremely rare case of LAA rupture by conventional aortic cross-clamp and repair of the same by fibrillating the heart with the aid of a Chitwood atraumatic vascular clamp.

## Case presentation

A five-year-old boy presented with complaints of palpitation, failure to thrive, and recurrent chest infections since childhood. On general physical examination, the patient was lying comfortably in the bed with a pulse rate of 120 beats per minute. Blood pressure was 96/60 mmHg of mercury and jugular venous pulse (JVP) was not raised. On cardiovascular system examination, S1 was loud. There was a fixed and wide splitting of S2. Grade 3 diastolic murmur was heard over the tricuspid area and Grade 3 ejection systolic murmur was heard at the left second intercostal space. On chest examination, bilateral air entry was symmetrical with a vesicular breath sound. The ECG revealed a normal sinus rhythm with a rate of 110 beats per minute. The P-waves were tall and peaked in lead II. The rSR pattern was present in leads V1 and V2. There was right axis deviation and right ventricular hypertrophy (RVH). Chest X-ray demonstrated plethoric lung fields with cardiomegaly, prominent main pulmonary artery, and right ventricular hypertrophy. Transthoracic echocardiography showed ostium secundum ASD of 15 mm size with a left to right shunt with an absent inferior vena cava (IVC) margin and three pulmonary veins draining into the left atrium (LA). No other cardiac congenital anomaly was present. The patient was referred for surgical closure of ASD.

**Figure 1:**
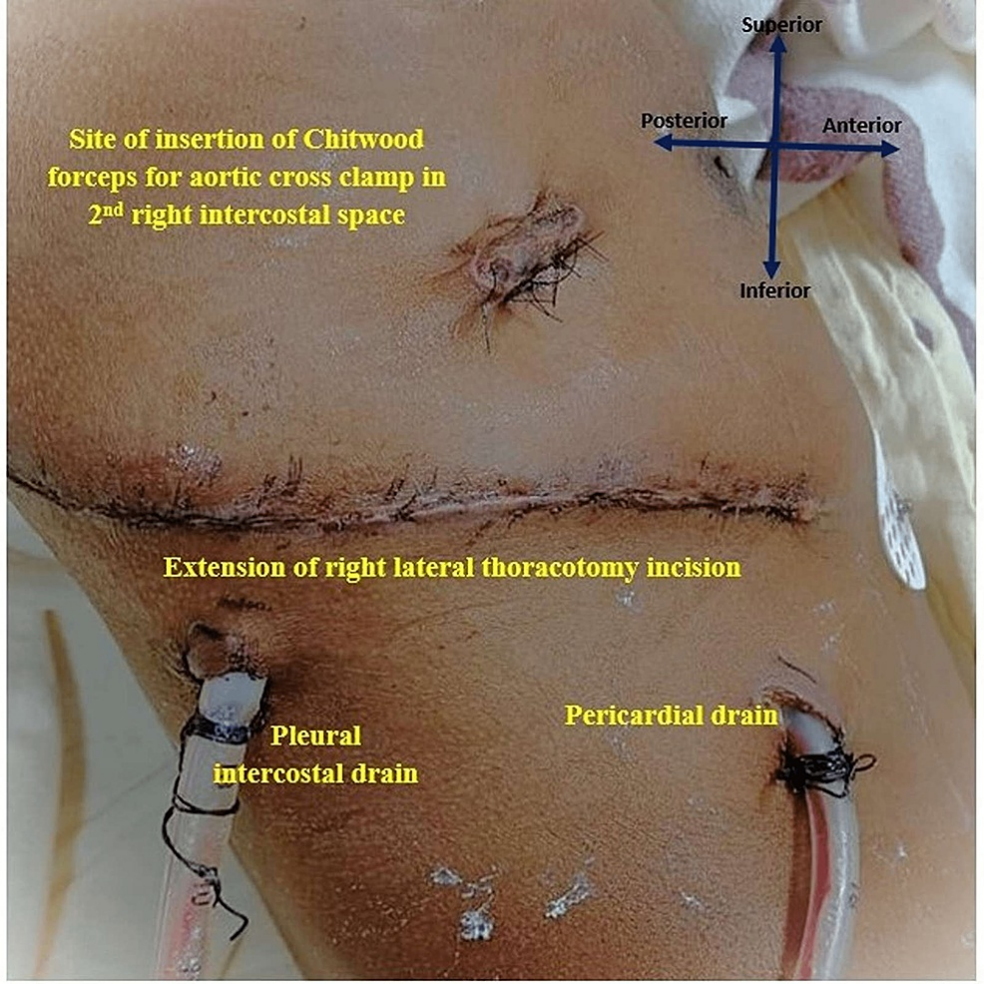
Post-operative image of the right anterolateral thoracotomy incision for left atrial appendage rupture closure with pleural and pericardial drain, and second intercostal space incision for insertion of Chitwood clamp.

**Figure 2:**
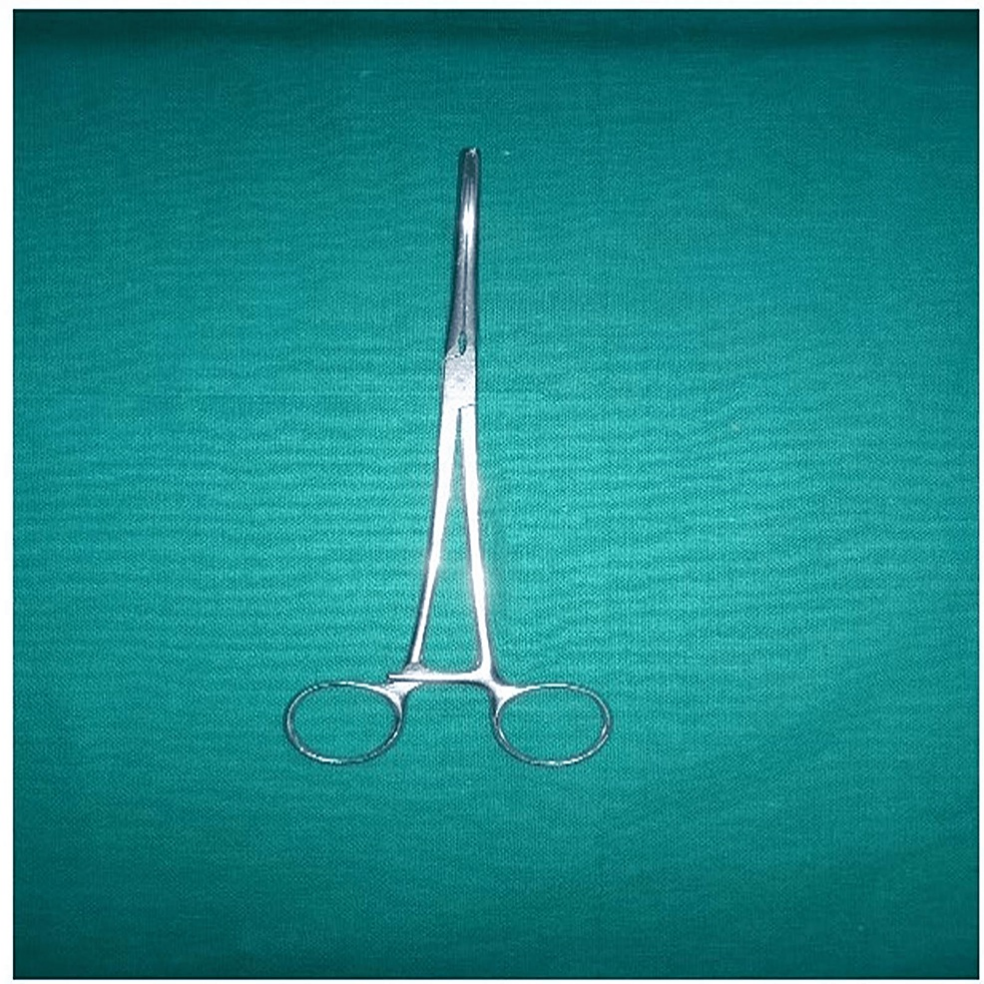
Conventional straight aortic cross-clamp

The patient was taken up for ASD closure through right anterolateral thoracotomy (Figure 1) under cardiopulmonary bypass with double lumen tube intubation and single lung ventilation (left lung). A curvilinear inframammary incision was made over the right fifth intracostal space. The chest was entered through the right fourth intercostal space. The pericardium was longitudinally opened 1 cm to 2 cm anterior to the right phrenic nerve and stay sutures were taken. Aortic and bicaval cannulation was done using an elongated aortic cannula (Figure 2)

**Figure 3:**
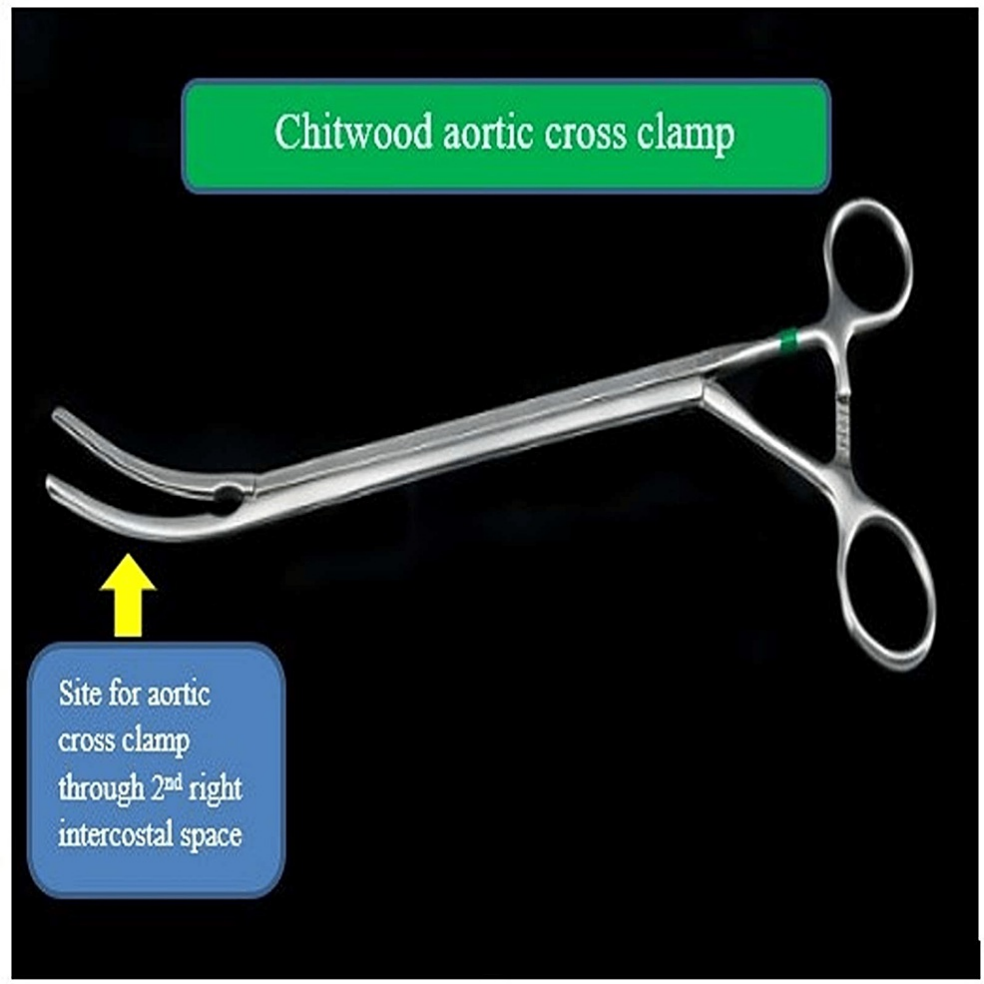
Chitwood aortic clamp Image created by the authors

**Figure 4:**
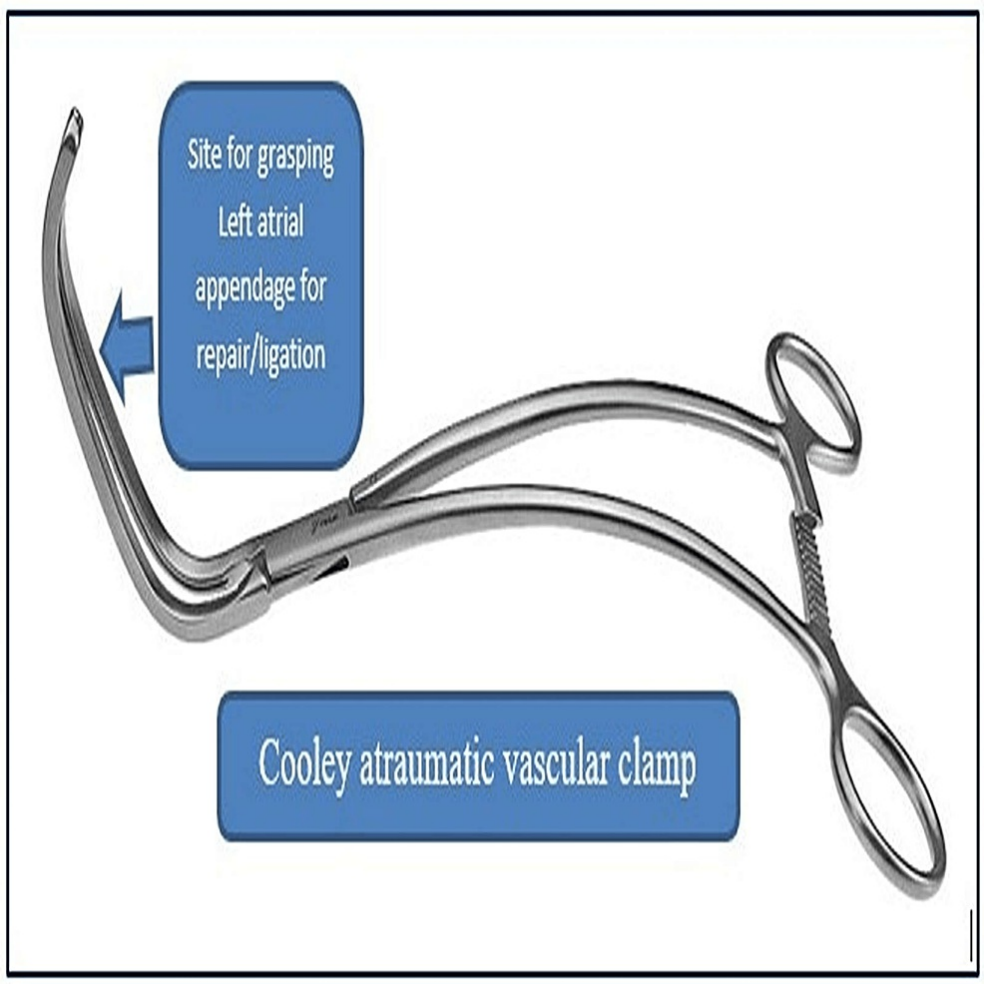
Cooley atraumatic vascular clamp Image created by the authors

Straight single cardioplegia cannula insertion was done in the ascending aorta. Umbilical tapes passed under the superior vena cava (SVC) and IVC. Aorta was cross-clamped using a straight aortic clamp and cold blood root cardioplegia was infused. Both SVC and IVC were snugged. The right atrium was opened. The ASD was closed with a pericardial patch. De-airing was done with an aortic needle vent. After the closure of the right atrium, caval tapes and aortic cross-clamp were removed. The patient was slowly weaned off from CPB. All cannulas were removed. Before the closure of the thoracotomy, it was found that the patient had continuous bright red-colored bleeding from the posterior surface of the heart. Measures were taken to localize the site of bleeding with proper evaluation, but failing which the right lateral thoracotomy incision was extended posteriorly and the heart was elevated for a few seconds, taking care to prevent arrhythmia (ventricular tachycardia and fibrillation) as the patient was already weaned from cardiopulmonary bypass pump and all decannulation were done. It was found that there was bleeding from the tip of the LAA. As the child was out of cardiopulmonary bypass (CPB), putting the child again on cardioplegia would have caused complications, hence it was decided to take the patient on CPB again after aortocaval recannulation and placing an aortic cross-clamp by inserting the Chitwood clamp (Figure 3) in the right second intercostal space in anterior axillary line after fibrillating the heart with a fibrillator temporarily without cardioplegia. Then, the injured LAA was held with a Cooley’s clamp (Figure 4) and the injury of about 0.5 cm of the LAA tip was repaired with prolene 5-0, and the LAA was tied with free silk 2-0. Then child's heart was defibrillated, the aortic cross-clamp was removed, weaned from CPB, and decannulation was done. Two drains were kept: one in the pleural cavity and another in the pericardial cavity (as seen above in Figure 1). The chest wall was closed in layers. The bypass time was 35 minutes and the cross-clamp time was 19 minutes Patient was extubated after six hours of surgery and was discharged after seven days. There were no ECG changes during the procedure and the bispectral index was also normal during the procedure.

## Discussion

The standard approach for most cardiac operations is median sternotomy. However, an unsightly midline scar can cause psychological distress, especially among young females. Anterolateral thoracotomy is one of the most frequently used incisions for ASD closure [1-5]. The reported complications of anterolateral thoracotomy include phrenic nerve injury [6-8], the collapse of the lung, infection, lung adhesion, scarring, and bleeding from the cannula insertion and atriotomy site. In our case, the patient had an unusual site of injury of the tip of the LAA which is not reported in the literature. The complication may occur while applying an aortic cross-clamp as in our patient. In such a scenario, the only option would have been converted to a median sternotomy approach to repair LAA injury, which would have defeated the idea of cosmesis. So we decided to extend the thoracotomy incision posteriorly so as to elevate the heart for ligating the LAA at its base. For repair of the LAA tip tear, the patient was taken on CPB without cardioplegia with the help of fibrillation and the application of a Chitwood clamp for the aortic cross-clamp inserted through the right second intercostal space in the anterior axillary line. So we suggest applying a Chitwood clamp for aortic cross-clamp during surgical ASD closure instead of a conventional aortic cross-clamp to prevent LAA injury [9-10]. Still, if there is any injury, it should be managed by fibrillating the heart temporarily under CPB without cardioplegia. The injury of the LAA should be repaired by 5-0 prolene and reinforced by 2-0 silk at its base for better hemostasis. We suggest the above maneuvers/techniques be adopted during ASD surgical closure or mitral valve replacement surgery by lateral thoracotomy approach in preventing any disastrous injury and subsequent management. 

## Conclusions

We report an extremely rare case of LAA injury during the closure of an ASD using a minimally invasive and cosmetic right anterolateral thoracotomy approach. Before the closure of the sternum, a vigilant look should always be there towards any unnatural side of bleeding like the LAA, which if missed would result in life-threatening intra and post-operative bleeding resulting in cardiac tamponade which would require urgent re-exploration adding morbidity and mortality to the patient, especially young children.
